# Mixed movement disorders revealing an atypical form of creatine deficiency syndrome

**Published:** 2015-01-05

**Authors:** Fahmi Nasrallah, Hanene Benrhouma, Ichraf Kraoua, Gilbert Briand, Souheil Omar, Ilhem Turki Ben Youssef, Naziha Kaabachi

**Affiliations:** 1 Department of Biology, School of Medicine, Laboratory of Biochemistry, Rabta Hospital, Jebbari, 1007 Tunis, Tunisia; 2 Department of Child and Adolescent Neurology, School of Medicine, Mongi Ben Hmida Institute of Neurology, 1700 Tunis, Tunisia; 3 Department of Biochemistry and Molecular Biology, School of Medicine, Laboratory of Endocrinology, Metabolism-Nutrition, Oncology, Biology Pathology Center CHRU, 57039 Lille, France; 4 Department of Biology, School of Medicine, Mongi Ben Hmida Institute of Neurology, 1700 Tunis, Tunisia

**Keywords:** Movement Disorders, Creatine Deficiency Syndrome, Inborn Errors of Metabolism

## Introduction

Creatine deficiency syndromes (CDS) are inborn errors of creatine (Cr) biosynthesis characterized by mental retardation and severe language impairment.^1^ Movement disorders, mainly dystonia have been described as additional features in CDS.^2^ We report on an exceptional case of mixed movement disorders due to an atypical form of CDS. A.H. is a 26-year-old man, born to second-degree consanguineous parents with family history of mental retardation in maternal cousin. Pregnancy and delivery were normal. Psychomotor development was normal. At the age of 6 years, he presented with a progressive cervical and left-hand abnormal posture with myoclonic jerks. When first examined at the age of 20, he had myoclonic jerks in the left upper limb with cervical and left hand dystonia. The diagnosis of heredodegenerative disease (inborn errors of metabolism) was evoked because of the consanguinity, family history, age of onset and mixed movement disorders. Oriented biological and imaging investigations were performed. Brain magnetic resonance imaging was normal. Serum copper level was 90 (Normal range: 80-160); urine copper level was 14 (Normal range < 20 μg/24 h); ceruloplasmin level was 0.260 (normal level: 0.2-0.6 g/l). Genetic testing for DYT1 gene was negative. Peripheral blood smear was normal. Amino acids and organic acids abnormalities and remethylation disorders were excluded. Urinary Cr and guanidinoacetate (GAA) were analyzed by gas chromatography–mass spectrometry; they showed low level of Cr associated with a relatively high GAA concentration and low Cr/GAA ratio (0.45) whereas a normal value exceeds 1.^3^ The diagnosis of mixed movement disorders due to an atypical form of CDS was made after the determination of intermediate GAA methyltransferase (GAMT) activity in lymphoblasts. Measurement of GAMT activity in lymphoblasts was performed according to Verhoeven et al.^4^

**Figure 1:**
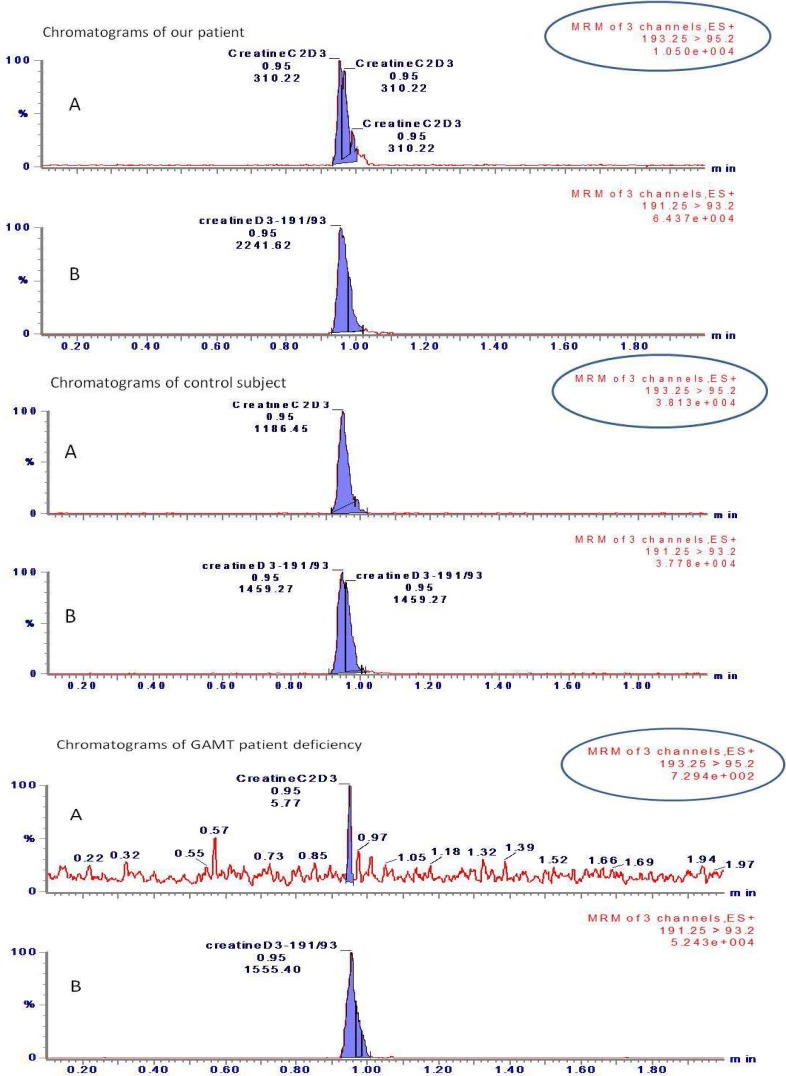
Chromatograms of patient (I), control subject (II) and guanidinoacetate methyltransferase patient deficiency (III) with an abundance = 1.05 e^+004^, 3.813 e^+004^, 7.294 e^+002^ respectively. (A; chromatogram of [^13^C_2_-^2^H_3_]-creatine and B; chromatogram of internal standard D3-creatine)

The clinical picture associated with the abnormal levels of Cr and GAA call the attention to CDS and particularly GAMT deficit for this patient. GAMT is the second enzyme in the process of Cr synthesis resulting from converting guanidinoacetate and S-adenosylmethionine into Cr and S-adenosylhomocysteine. Patients with GAMT deficiency exhibit complex clinical phenotypes with hyperkinetic movement disorders such as generalized dystonia and severe mental retardation with epilepsy.^1^ Though reduced the GAMT activity in this patient, which was 0.107 nmol/h/mg protein (normal values: 0.29-0.31 nmol/h/mg protein), is equivalent to that reported for heterozygous parents.^5^ As shown in figure 1, the GAMT activity of this patient was intermediate between that of the control subject and patients with a total GAMT deficiency, it was below the detection limit in GAMT deficiency patient (< 0.01 nmol/h/mg protein). Two hypotheses could be proposed to explain the association of a low Cr/GAA ratio with a partial deficit of GAMT activity and a clinical picture characterized by the presence of mixed movement disorders. The low Cr/GAA ratio could be attributed to a high endogenous consumption of Cr originating partly physiologically (50%) from high meat nutrition and partly (50%) from body synthesis. As for the partly deficient GAMT activity, it could be an atypical form of CDS with a non-ubiquitous GAMT deficiency. Additional explanations and hypothesis would be advanced once similar cases are studied.

## Notes

**How to cite this article:** Nasrallah F, Benrhouma H, Kraoua I, Briand G, Omar S, Turki Ben Youssef I, et al. Mixed movement disorders revealing an atypical form of creatine deficiency syndrome. Iran J Neurol 2015; 14(1): 47-9.
